# Directed causal effect with PCMCI in hyperscanning EEG time series

**DOI:** 10.3389/fnins.2024.1305918

**Published:** 2024-04-15

**Authors:** Lykke Silfwerbrand, Yasuharu Koike, Pär Nyström, Malin Gingnell

**Affiliations:** ^1^Department of Medical Sciences, Psychiatry, Akademiska Sjukhuset, Uppsala, Sweden; ^2^Institute of Innovative Research, Tokyo Institute of Technology, Yokohama, Japan; ^3^Department of Psychology, Developmental Psychology, Uppsala University, Uppsala, Sweden; ^4^Department of Psychology, Division of Emotion Psychology, Uppsala University, Uppsala, Sweden

**Keywords:** dual-EEG, hyperscanning EEG, causal effect, directed causality, PCMCI, Tigramite

## Abstract

Social activities are likely to cause effects or reactivity in the brains of the people involved in collaborative social situations. This study assesses a new method, Tigramite, for time domain analysis of directed causality between the prefrontal cortex (PFC) of persons in such situations. An experimental situation using hyperscanning EEG was applied while individuals led and followed each other in finger-tapping rhythms. This structured task has a long duration and a high likelihood of inter-brain causal reactions in the prefrontal cortices. Tigramite is a graph-based causal discovery method to identify directed causal relationships in observational time series. Tigramite was used to analyze directed causal connections within and between the PFC. Significantly directed causality within and between brains could be detected during the social interactions. This is the first empirical evidence the Tigramite can reveal inter- and intra-brain-directed causal effects in hyperscanning EEG time series. The findings are promising for further studies of causality in neural networks during social activities using Tigramite on EEG in the time domain.

## 1 Introduction

Social activities are a key part of life. These activities, whatever they are, have an element of causing effects or reactions in other people involved. Social neuroscience traditionally focused on one brain at the time to understand the social side of the humans but is recently employing hyperscanning technology to include at least two persons in social interactions (Babiloni and Astolfi, 2014; Pan et al., 2022). More and more, the technology has made it possible for two-person, or more, neuro science measuring neuronal reactivity during social behavior (Hari and Kujala, 2009; Dumas, 2011). This approach opens up to further understand how individuals influence each other during bi-, or multi-directional information exchange (Hasson and Frith, 2016).

Social interactions between individuals entail the activity of one person and the prediction of what the other person will do, followed by the other’s action and a correction of the original subjective prediction (Frith and Frith, 2010; Vesper et al., 2010; Takai et al., 2023). In predicting and synchronizing with a partner this two-person activity has internal (intra) and external (inter) brain components. For obvious reasons, the inter-brain components have no factual neural connections.

Social behavior is often expressed as motoric activity in moving together in synch with one another. Preparation for motoric synchronization with another person’s expected actions followed by correction of what happened has been described as an internal forward model for motoric behavior, combining a predictive forward model with an inverse model that refines goal-directed action (Kawato, 1999; Ganesh et al., 2014). This internal model was developed in relation to movements but can be generalized to what is called predictive coding of the brain concerning all sensory modalities (Friston and Kiebel, 2009). The cognitive element of predictive coding of the brain is assumed to be situated in the pre frontal cortex (PFC) (Tanaka et al., 2020). Predicting behavior has also shown that the PFC is active in processing external information (Kawato, 1999; Karnath, 2001; Frith and Frith, 2010; Rosazza and Minati, 2011; Abreu et al., 2012). Interactive steps combining the preparation, prediction and adaptation could continue to take turns and eventually amount to continuous adaptation or synchronization of the involved individuals. Responses and adaptations could thus be based on the cognitive predictions and subsequent responses to the partner’s actions (Tanaka et al., 2020). The PFC, together with the temporal parietal junction, is also described as a part of the mutual attention system (Gvirts and Perlmutter, 2020). It is perceived that the higher the inter neural synchronicity in the social attentions systems, the higher the tuning in between the participants. Coupling of this system between individuals may help increase in tuning with one another for communal interaction goals (Anders et al., 2011; Gvirts and Perlmutter, 2020). Hyperscanning brain imaging studies have shown frontal–frontal synchrony during such human affiliation (Hu et al., 2017; Reindl et al., 2018; Schwartz et al., 2022). Factors that facilitate persons to neurally synchronize are face-to-face settings so that the partaking individuals can see each other and interact (Gvirts and Perlmutter, 2020). Another factor is that the social activity evokes engagement in the participants. Joint, social activities activate areas in the PFC, especially activities that are not competitive. The nature of the interacting partner also influences the synchronization. Friendly cooperation favor the activity in the mutual social attention systems (Gvirts and Perlmutter, 2020).

Music and rhythms have emerged as promising methods to find ecologically valid experimental situations for hyperscanning concurrent brain imaging of at least two participants during social behaviors (D’Ausilio et al., 2015; de Reus et al., 2021). Rhythms, mostly tapping, have been widely used to investigate interactive tasks (Repp, 2005; Repp and Su, 2013). A setup that allows participants to mutually collaborate is to alternate between leading and following in finger-tapping rhythms (Silfwerbrand et al., 2023). Hyperscanning is necessary to capture the entire dynamics of the combined systems, such as when two or more individuals simultaneously are participating in an experiment (Czeszumski et al., 2020). A setting that offers face-to-face communication is with hyperscanning EEG, when the participants are able to sit and interact with each other in the same room, rendering an ecologically valid situation for significant interaction of the mutual social attention systems of the brains (Gvirts and Perlmutter, 2020). In such a setting activation of the PFC in the mutual social attention system could be expected based on shared intentionality and mutual goals.

Analyses of synchronous brain activity are typically using the time domain for correlation analyses, or frequency domain estimating synchronous activity (Astolfi et al., 2010; Dumas et al., 2010; Schippers et al., 2010; Babiloni and Astolfi, 2014). While frequency based connectivity estimation is commonly used for neuroelectrical hyperscanning, temporal correlation and Granger causality is usually applied on heamodynamic hyperscanning, such as fMRI and fNIRS. EEG has low spatial resolution, but high time resolution. Hence, a time domain analysis of hyperscanning EEG time series could give relevant estimations of causal connectivity between individuals. A newly developed PCMCI algorithm for detecting causal links in geophysical data has been used on fMRI (Runge, 2018a; Runge et al., 2019b; Saetia et al., 2021). This is a combination of the Peter and Clark (PC) algorithm for finding influencing data to every time point and an algorithm for momentary conditional independence (MCI) (Mäkelä et al., 2022). Applying the PCMCI algorithm on EEG data for causal connectivity could show if synchronous activation of two mutually interacting individuals also indicate directed causality.

Hyperscanning in useful in accessing both intra- and inter-brain connectivity. Causal connectivity is directed connectivity in which a sender of information causes a reaction at the receiver end of the connection. Directed connectivity of brain areas is commonly analyzed as functional neuronal connectivity (Friston et al., 2013). Functional neuronal connectivity refers to the statistical dependence or common information of neuronal systems, measured in physiological responses from one point to another (Schmälzle et al., 2017). This is commonly analyzed using Granger causality (Granger, 1969; Seth et al., 2015). Functional connectivity tests whether there exists a dependency between two or more time series. Granger causality is testing if there is a statistical dependency of a time point with measured activity in the past (Friston et al., 2013; Seth et al., 2015) So, Granger causality gives directional causal relationship if the past values of one time series (A) has information to describe future values of another time series (B) better than the past values of B does by itself.

A complementary approach to Granger causality for finding causality in time series data is Tigramite. Tigramite applies the PCMCI algorithm and combines finding directly influencing data points to every time point using the Peter and Clark (PC) algorithm with calculating the momentary conditional independence (MCI). It also gives the direction of causal connections between time series, but accepts non-linear data sets and data with large number of variables compared to the number of data points (high dimensional data) (Runge et al., 2019b). An initial test of correlation is made before applying PCMCI, to decide the number of time steps to include in the analysis. The correlation of activity at each time point to all previous time points is assessed. The time point representing the point of decaying correlations will be used in the PCMCI algorithm. This means that the PCMCI algorithm will describe the causal connections of, say three, time steps preceding each time point. The first step (PC) of the algorithm is aimed at finding a model with the smallest number of connections that represent the causality of the time series within the chosen time frame. The second step (MCI) is controlling for false positives in the causal network resulting from the first step (Runge et al., 2019b). This two-step approach maximizes the detection of causality and minimizes the number of false positives (Runge, 2018b). This method, which is graph-based, considers indirect links and common drivers of links and has a high detection of actual causality while minimizing false positives (Runge et al., 2019a).

The PCMCI algorithm is here applied to a hyperscanning EEG time series of the PFC of three pairs interacting through finger tapping. This set up allows for assessing causal neural reactivity of the social attentions systems expressed as activation of the PFC during the cooperative social activity. The PCMCI may be the appropriate choice here since it is applied at the time series data resulting from the PFC reactivity of the individuals involved, rather than using frequency analysis (Krich et al., 2020; Mäkelä et al., 2022). The setting facilitates mutual social attention with letting two people face each other sitting on chairs with small a table between them. They are engaged in the collaborative task of taking turns in leading each other in finger tapping simple rhythms. The hyperscanning EEG system lets them to sit relaxed, creating an ecologically valid social situation.

This study aims to show if PCMCI, the graph-based causal discovery method, can be applied for time domain causal analysis of hyperscanning EEG data.

## 2 Materials and methods

To test the suggested PCMCI algorithm the most relevant part of a data set of hyperscanning EEG was applied. The complete data set is described in a forthcoming article.

### 2.1 Participants

Eight right-handed persons, one female, [mean age 31.7 years (SD±8.1)] participated in the study. All participants reported being healthy, without formal musical education and no documented professional leadership expertise. Participants were a convenience sample recruited from a technology university in Tokyo. Each participant volunteered and gave their written consent to participate in the study. All procedures were in accordance with protocols approved by the local institutional review board (dnr. 2,019,002). Processing of the data in Sweden was approved by the Swedish Ethical Review Authority (dnr. 2021-05481-01). The data from one participant pair was excluded due to noise (as assessed by visual inspection). After the exclusion of the data, six participants (one female) remained for analysis.

### 2.2 Questionnaires

After the experiment, the participants rated their experience of leading and of following on a scale from 1 (poorly) to 5 (successfully).

### 2.3 Setup

The participants sat facing each other with a small table between them. They were equipped with EEG caps of 64 BioSemi® electrodes (BioSemi instrumentation). The right hand was on the table where they tapped the rhythms with the right index finger, the left hand resting on their thigh. The movement of the right index finger was recorded with motion caption system sensors, Optitrack (NaturalPoint, Inc).

### 2.4 Tapping together

**Figure 1:**
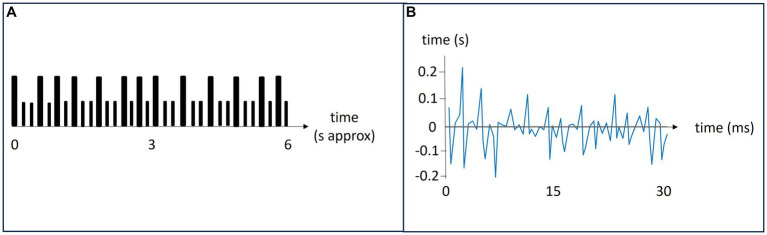
Panel **(A)**: A schematic representation of a rhythm. Large bars indicate a tap and small bars indicate silence. The rhythms lasted about 6 s and were repeated 5 times during each task. Panel **(B)**: A representation of the relative synchronicity in tapping. The leader’s tapping is the baseline. It is here represented by the x-axis. The follower is tapping along with the leader, and their difference in tap timing is shown in blue.

Each participant received an audio file with an easy-to-learn rhythm to remember by heart the day before the experiment. The rhythms were similar, yet unique, per participant. During the study, the pairs were tapping their respective rhythms with each other taking turns to be the leader. Figure 1A depicts a schematic representation of a 6 s rhythm that was repeated five times per task. The tasks were to lead in tapping the pre-learned rhythm, tap alone in one’s pre-learned rhythm, follow in the other’s rhythm and rest (do nothing). There were two session consisting of four blocks with four tasks. Instructions of what to do were delivered vocally as the study unfolded.

### 2.5 Synchronicity in tapping

For all participants, double taps (taps interspaced with <0.2 s) and extreme outliers (inter-tap intervals >2 s) were excluded from the analysis. Both double taps and extreme outliers were rare, in most sessions there were none. For each task, the string of taps of the leader was used as a model for the correct rhythm. The taps of the follower were then matched to this rhythm and the relative difference between each tap was calculated. Thus, synchronicity was defined as the time difference between the tap of the leader and follower, i.e., the relative adaptation of the follower to the leader’s tapping rhythm. The typical mean reaction time from hearing a sound to finger tapping was 0.16 s (Kosinski, 2010; Woods et al., 2015). Figure 1B shows a follower’s temporal synchronicity with the leader, depicted by the blue line. The leaders tapping sets the baseline, represented by the x-axis.

### 2.6 EEG data acquisition

Two 64-channel BioSemi® systems were interconnected via an LSL (Lab Streaming Layer) for synchronization. The EEG data were collected continuously during the tapping sessions with a sampling rate of 4,096 Hz.

### 2.7 Data selection for causal analysis

To focus on activity and reactivity in the PFC within and between participants, data from the three electrodes representing the PFC were used. These were F3 (Brodmann area, BA 9 in the left hemisphere), F4 (BA 9 in the right hemisphere) and FZ (BA 32).

### 2.8 EEG data first-level processing

EEGLAB (Delorme and Makeig, 2004) and MoBILAB (Mobile Brain/Body Imaging Lab) (Ojeda et al., 2014), an EEG plugin for synchronized in-data, were used for data preprocessing. Both software systems ran on Matlab 2018b (Mathworks, Inc). For the location of the EEG electrodes, BESA® (Brain Electrode Source Analysis, GmbH) channel positions were used. The reference was the average of the 64 active electrodes. Preprocessing followed the EEGLAB documentation, starting with examining the data for outliers, gliding and extreme channels (Delorme and Makeig, 2004). Few passages per task were removed in this process. A high-pass filter of 1 Hz was used to remove slow drifts from the data. No low-pass filter was applied. The data were resampled to 100 Hz and event times for “lead” and “follow” were registered. As a last preprocessing step, independent component analysis (ICA) “runica” was applied to each dataset to extract statistically independent components (IC). The IC that by EEGLAB were labeled muscle, eye and line components of at least 90% accuracy were removed from the set. About 15 IC per task were removed in this step. Due to some lasting movement and muscle related activity as the 64 channels were recalculated, runica was rerun and the data were repruned using the same rules, following the EEGLAB documentation (Delorme and Makeig, 2004). In the second round minimizing noise, about 5 ICs were removed per task. After preprocessing, the three electrodes representing PFC, F3, Fz and F4, were extracted for the second level analysis.

### 2.9 PCMCI analysis

Preprocessed EEG data that had been removed of noise and artifacts and separated into time series per activity of interest, were analyzed paired-wise for directed causality. The open-source Jupyter Notebook for Tigramite was used for the causal analysis (Runge et al., 2019b). Software used were Python 3.8.5, by Python Software Foundation and Jupyter (Kluyver et al., 2016). This two-step analysis of Tigramite has been shown to have high detection power and low false positives compared to other tools (e.g., Granger causality) for connectivity analysis (Runge et al., 2019a). This approach comes with a set of assumptions. Basic assumptions for the time series used are that they are stationary, conditionally independent and without missing values. Stationarity assumes that the statistical properties for the EEG timed series, such as the mean and variance, do not change over the time of each task. The data should not be too volatile for the period of interest. The independence assumption is needed for the test of conditional independence and implies that all the information of one time point has of another time point can be present in a third time point. This third time point can hold causal information or be a mediator in the causal path between the first and second time points. In an EEG time series it is plausible that some time series that are partially independent such that a causality path is involving more than one time point. This indirect relation can be assessed in the second step of PCMCI. No missing values are also assumed for the time series, so that all values are represented in the EEG time series with no false or null values.

There are also three main assumptions for causal relations in this graph-based analysis of measured time series: causal sufficiency, causal Markov condition and faithfulness (Runge, 2018a). Causal sufficiency assumes that no other unobservable variable changes these variables directly or indirectly. Causal sufficiency is required because it is impossible to ensure that all variables are measured in an EEG data collection. The causal Markov condition implies that, when the value of a node’s predecessor is known, no other variables become relevant for predicting the state of the current node. This can be assumed for EEG time series since the data collected is assumed to represent the relevant brain activity of the situation of interest. The main assumption, faithfulness, guarantees that the current graph holds all conditional independence relations implied by the Markov condition (Spirtes et al., 2000; Glymour et al., 2019). This can also be assumed for EEG time series data, since the data collected represents the relevant brain activity of the situation that is under analysis. Causal interpretation also assumes stability, to ensure that the observed conditional independencies in the data accurately reflect the underlying causal structure, not some artifacts or coincidental cancelations. In EEG time series it is of importance to preprocess the data so that the resulting time series actually represents the neuronal activity of interest and not noise or some coincidental activations.

**Figure 2:**
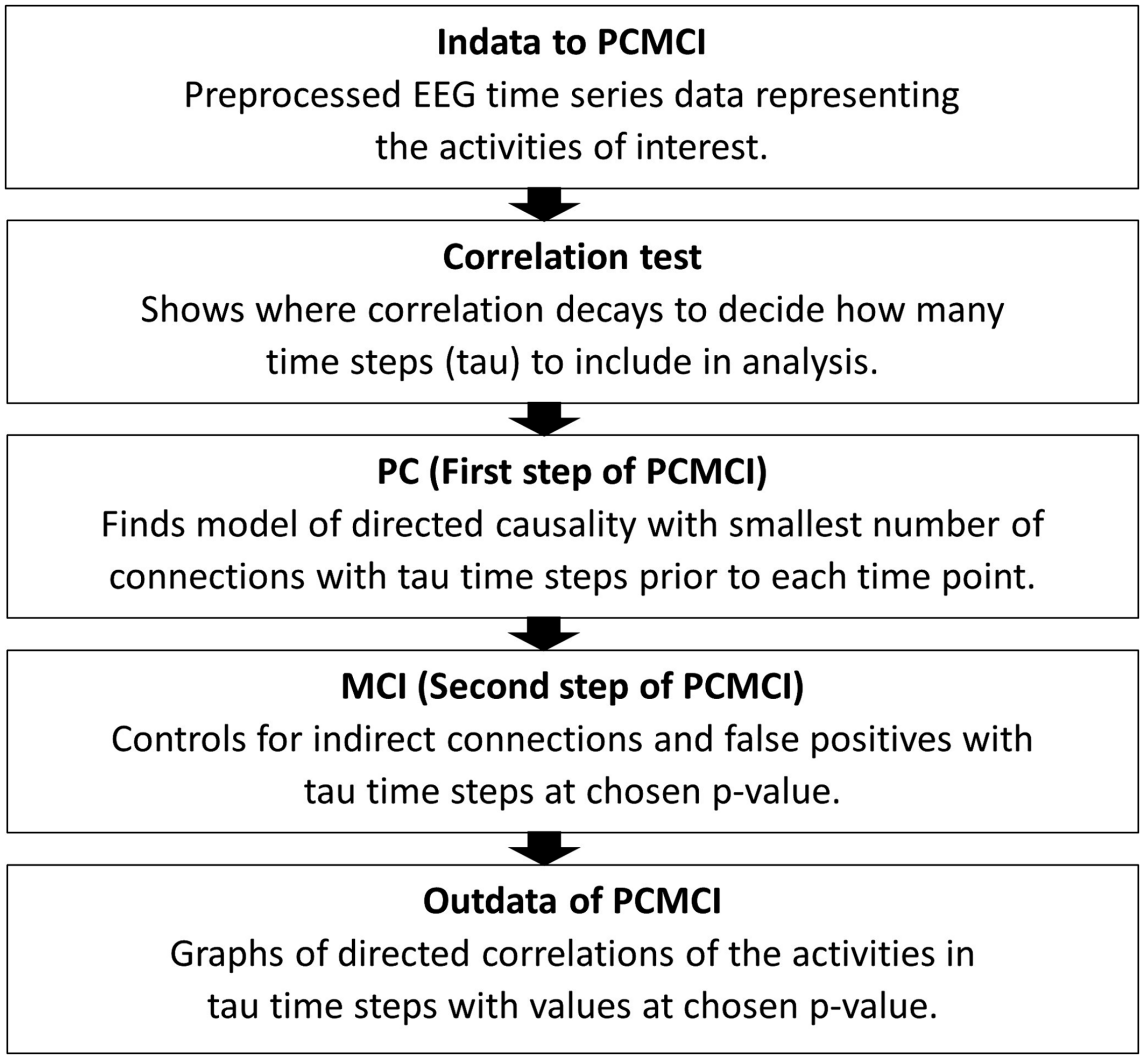
A schematic summary of the steps to apply PCMCI to EEG time series data. Indata is time series data separated into the activities of interest. In this case pair-wise activities were assessed for correlations, matching leader time series EEG data from one person with the following time series EEG data from the other person and vice versa. After the correlation test tau is chosen and in the third step a p-level (FDR) is chosen. The final result comes in a process graph and a time series graph of tau steps with values for the correlations between the activities entered in to the PCMCI algorithm.

The first step of analysis, before applying the PCMCI algorithm, is a correlation test to find out the number of time steps to include in the PCMCI. For datasets such as EEG, with assumed linear interdependencies, the partial correlation method of ParCorr for conditional independence test is used (Runge, 2018b). Connections between brain areas in EEG data series can be assumed linear, even though anatomical structures can differ between individuals (Friston et al., 2013). The correlation test shows how many time steps before each time point of activity in the EEG data are influencing, or correlating with, at a data point. This results in a graph showing the decaying of the correlations. Tau, the number of time points included in the analysis, is the point in time where the correlations are closing zero. This time point, tau, will be used in applying the PCMCI algorithm so that it describes the causal connections of tau time steps in the time series. The first step (PC) of the algorithm is using the Akaike Information Criterion (AIC) to find the model with the smallest number of connections which represent the directed causality of the time series within tau time steps. Momentary Conditional Independence (MCI), the second step of this algorithm, is controlling for indirect causal connections and false positives in the resulting causal network. This final step assesses indirect causation and detects false positives for every time point. It tests, at a chosen p-level (FDR), if a set of time points removes the association between two other time points detecting false positives (Runge, 2018a). See Figure 2 for a schematic representation of these steps.

## 3 Results

Two situations are analyzed in this study. Connectivity is measured between the three EEG electrodes (F4, Fz and F3) per brain. In addition, the connectivity between the same electrodes in two communicating brains is measured in the inter-brain analysis.

### 3.1 Tapping synchronicity and questionnaires

The mean relative synchronicity between leaders and followers was 0.0065 s (SD = 0.021 s). The participants reported comfort levels leading (mean = 3.3) and following (mean = 3.2).

### 3.2 Causal analysis

**Figure 3:**
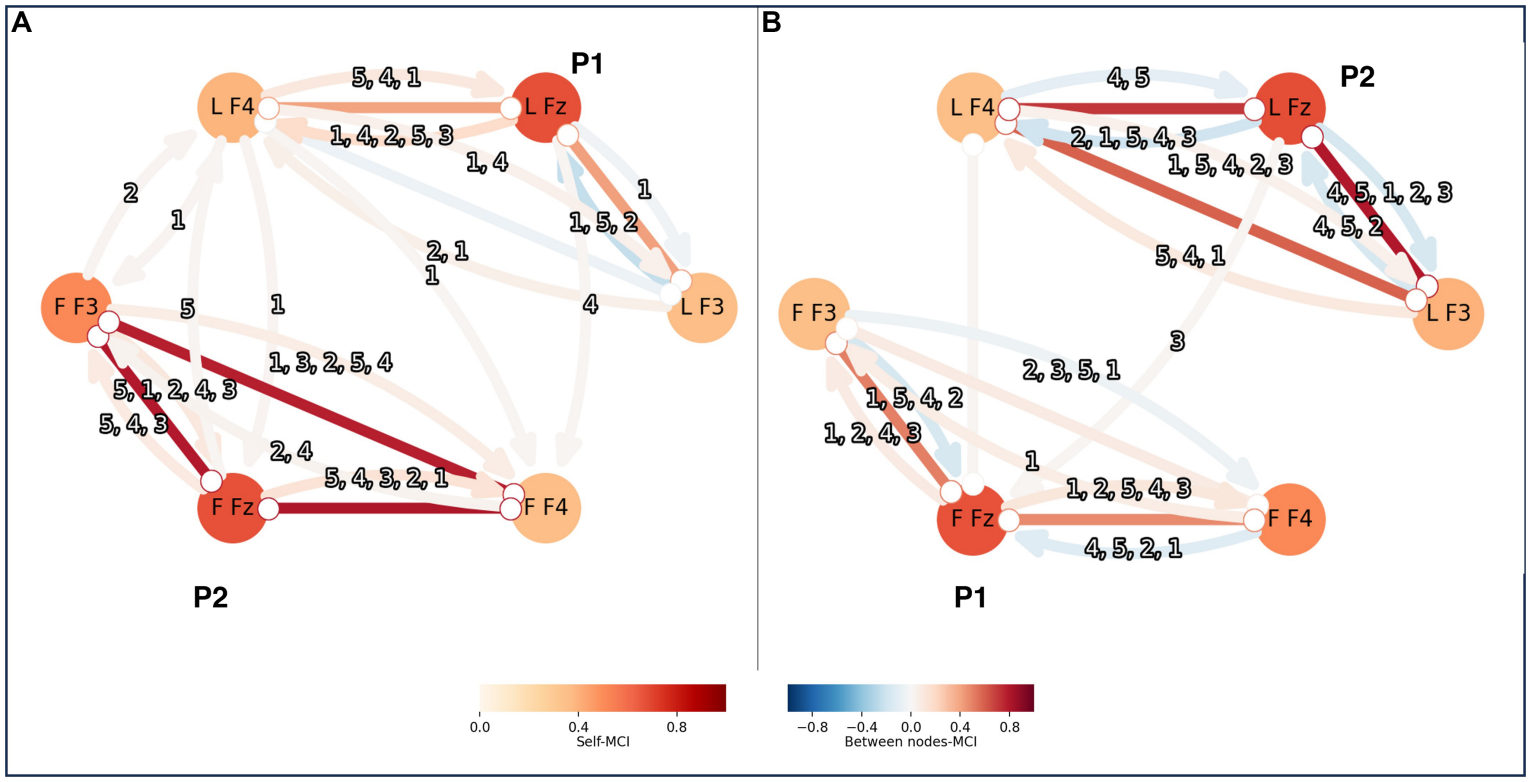
PCMCI was calculated both within persons and between pairs of leader-follower. The result is shown here as nodes and connections, with stronger colors for stronger correlations. Each person’s brain is represented by a triad of EEG-electrodes: F4, Fz and F3. Here is an example of two persons, P1 and P2, forming a pair. In panel A P1 is the leader and P2 is the follower, and in panel B P2 is the leader and P1 is the follower. The triad of the leader has the letter L in front of the EEG-electrode names and the triad of the follower has the letter F in the same place. The connections within a triad of EEG-electrodes reflect the PCMCI correlations within a brain. The connections between triads represent PCMCI correlations between the pair. The numbers indicate the time steps of the existing correlations, starting with the one that had the strongest correlation. For example, “4,5,1,2,3” when P2 was leader there were 5 correlations from L Fz to L F3 within the brain. The strongest correlation was from 4 time steps before the time point of interest, this is here shown as a blue arrow from L Fz to L F3. Other correlations existed, with lower and falling values at 5, 1, 2 and 3 time steps leading up to the time point of interest.

Visual inspection of the time series data imply that they are stationary and without missing values. To choose the maximum time lag (tau), or how many time steps to include, in the causal analysis, partial correlation (ParCorr) of the included time series was calculated. Two time series at the time were measured, one from a leader and one from a follower. The lagged dependencies between the two time series were overall very small and within individuals they were decaying after around 5 time steps. Thus, tau of 5 was applied for the PCMCI calculations. The EEG time series were of 12,400 data points. They were conditionally independent sufficiently for the independence test of Tigramite. The PC algorithm was applied aiming to remove all irrelevant conditions for causality, with an alpha parameter that was optimized to 0.05 by PCMCI using the AIC. The following MCI algorithm attempted to remove lasting false positive links. For the MCI the *p*-value was set to 0.05 (corrected for multiple testing by controlling the false discovery rate (FDR) using the Benjamini-Hochberg algorithm). The resulting PCMCI statistic can be interpreted as the causal link from one node to another within a network. Nodes earlier in time, can influence, or causate, a node at a time point. In Figure 3 an example of the result the two setups analyzed here is shown, one setup is for connectivity within one brain and one is for connectivity between two brains.

### 3.3 Within-subject causal relations

The significant intra-brain causal relation in all directions is recorded between all electrodes with tau = 5 and an FDR alpha of 0.05. The absolute values ranged between 0 and 1 (mean = 0.28, SD = 0.20). In Figure 3 shows a graph of the causal relations between the three EEG electrodes representing the PFC. LF4, LFZ and LF3 represent the electrodes on the leader and FF3, FFZ and FF4 are the electrodes on the follower. The color of the nodes, the self-MCI, refer to causal relations with the same electrode and between nodes-MCI refers to causal relations between electrodes. Relations with arrows depict causal directions. Relation numbers indicate the number of steps back that the relations originate. With tau = 5, the relations are traced back up to 5 time steps per electrode (Figure 3 shows an example graph of causal relations).

### 3.4 Between-subjects’ causal relations

Significant inter-brain relations are recorded for pairs time series with tau = 5 and FDR *p* = 0.05. For one pair there was significant connection only when one of them was leading, not when the other one was. The PCMCI values ranged from absolute 0.015 to 0.025 (mean = 0.036, SD = 0.017). The connections between subjects are presented as arrows between the leaders and followers in Figure 3. The relations are directed from leader to follower or vice versa. The total number of causal relations from leader to follower were 21 (mean per person was 3.5, SD = 2.2) and 13 from follower to leader (mean = 2.2, SD = 2.7). See Appendix A for all values.

## 4 Discussion

Significant directed causal connectivity of the PFC was shown between all pairs during the social interaction of collaborative in finger tapping rhythms together. There was also significant directed causal connectivity within the PFCs of all participants, indicating that the PFC was active for all during this social activity. The causal connectivity between the brain areas representing social connectedness, attention toward other people and cognitive predicting of other’s activities indicates that using PCMCI can be efficient in reporting directed causality in hyperscanning EEG experiments.

This experimental hyperscanning EEG study measures causal effects using the Tigramite graph-based causal discovery method of PCMCI (Runge et al., 2019b). Causal effects are measured within and between regions of the PFC expected to be active during socially engaging activities and a collaborative setting (Repp and Su, 2013; Hu et al., 2017; Gvirts and Perlmutter, 2020; Kim, 2020). The relative temporal synchronicity between leader and follower was close, indicating that it was fairly easy to both lead and follow, whereas the relatively high variance shows that there were differences in timing, which can be due to the time it takes for the follower to initially learn the rhythm and then continuously adapt to the leader’s tempo. This could represent any socially engaging situation where adaptation, or synchronization, is ongoing. The questionnaires indicated fairly high-rated degrees of success for both roles. This implies that the situation was engaging to a degree and that there was collaboration to reach a common goal (tap the rhythm) was present. These findings suggest participants were mutually socially attentive, that they were tuning in on and predicting the others actions (Friston and Kiebel, 2009; Gvirts and Perlmutter, 2020; Tanaka et al., 2020; Pan et al., 2022).

The inter-brain causal effect does not show hard-wired physiological information but could reflect how the brains not only synchronize, but causate each other, in their activities. Predictive coding and mutual social attention have previously been related to synchronized brain activity in the PFC (Frith and Frith, 2010; Sacheli et al., 2013; Koban et al., 2019; Gvirts and Perlmutter, 2020). Here, directed casual activity during engaging social collaboration is also shown. Predictive coding of the two participants may also include predicting the other’s actions as a part of the continuous directed causal effect detected in the PFC of both leader and follower (Den Ouden et al., 2005; Kim, 2020). This between-brain causal effect could reflect the synchronicity or adaptation to the other’s activity. Interpreting the specific value of the causality and whether it is relevant to raise the issue of higher or lower causality in EEG data is a question for future studies (Granger, 1969; Babiloni et al., 2007; Runge et al., 2019b; Cometa et al., 2021). For this study, the existing significant directed causal effect within and in both directions between leader and follower is interpreted as a continuous mutual causation.

The causal effect can be interpreted as influencing the state of another entity (Granger, 1969; Cometa et al., 2021). The causality has directions representing the direction of this influence between entities (Schippers et al., 2010; Friston et al., 2013; Cometa et al., 2021). Measuring such causality in neuroimaging data has been attempted by different methods. In EEG data Granger causality have most often been applied for causal connectivity (Dumas et al., 2010; Kawasaki et al., 2013; Babiloni and Astolfi, 2014; Czeszumski et al., 2020). Granger causality test whether one time series is preceding and predicting another, based on that it contains past values that can help predict the other time series (Seth et al., 2015). Granger causality does not account for indirect links or common drivers of causations like PCMCI, which may be relevant in neuronal networks (Runge et al., 2019b; Saetia et al., 2021). PCMCI uses the causal Markov condition and faithfulness assumption, which is a crucial base for graphs marking directions for causal relationships (Lauritzen and Richardson, 2002). PCMCI can account for confounding factors by testing the conditional independence given other observed variables. It also results in a graph that is useful for visualizing and understanding complex interactions in the data (Runge, 2018a).

There may be other drivers of the causality than described in any dataset trying to depict complex real-world data, such as neuronal reactivity (Runge et al., 2019b). Causality with other drivers than what was measured in these time series, direct or indirect, would mean that more causal reactions occur than shown here (Runge et al., 2019b). False positives in the result would give slightly more reactions than present in the dataset (Runge, 2018a). Such complementary drivers would not change the interpretation of the result much because this method has comparatively high detection power and a low number of false positives (Runge et al., 2019a; Saetia et al., 2021).

To set up a socially engaging situation in an experimental setting can be a challenge. Here two people were introduced and sat facing each other across a table. Both wearing EEG caps of 64 channels that were connected to a computer. They could move around, but were asked to keep as still as possible for the best possible quality of the EEG signals. This environment is possibly not inducing engagement, still all participants engaged in the finger tapping together with the person opposite the table. This scenario came close to a possibly engaging social situation of mutual engagement.

A limitation of the dataset is the low number of participants. There was also a variation of a causal effect between participant pairs, and it is not evident how our findings generalize to a broader population. However, because all pairs had significantly directed causality within and between brains makes it likely that our results are robust. Further studies should recruit a larger sample to ensure the generalizability of the result.

## 5 Conclusion

This study is an empirical support that hyperscanned EEG can show both inter- and intra-brain causal effects using the graph-based causal discovery method of Tigramite. Significant directed causal effect within and between the PFC was demonstrated during a collaborative social task. Despite the small sample size, all be it with significant causal results in all tests, the results are promising for future causality hyperscanning EEG studies using PCMCI.

## Data availability statement

The raw data supporting the conclusions of this article will be made available by the authors, without undue reservation.

## Ethics statement

The studies involving humans were approved by Tokyo Institute of Technology review board, dnr. 2019002; Swedish ethical review authority, dnr. 2021-05481-01. The studies were conducted in accordance with the local legislation and institutional requirements. The participants provided their written informed consent to participate in this study. Written informed consent was obtained from the individual(s) for the publication of any potentially identifiable images or data included in this article.

## Author contributions

LS: Conceptualization, Data curation, Formal analysis, Investigation, Methodology, Project administration, Software, Writing – original draft, Writing – review & editing. YK: Conceptualization, Methodology, Resources, Software, Supervision, Validation, Writing – review & editing. PN: Methodology, Supervision, Writing – review & editing. MG: Conceptualization, Data curation, Formal analysis, Methodology, Supervision, Validation, Writing – review & editing.
