# Supplementary material for: Directed causal effect with PCMCI in hyperscanning EEG time series
Source: Front Neurosci. 2024 Apr 15;18:1305918. doi: 10.3389/fnins.2024.1305918 (PMC11057377; doi:10.3389/fnins.2024.1305918)
Supplement: Supplementary file 1 [file Table_1.DOCX]

Appendix A

Summary statistics of PCMCI connections between participants with tau=5 and p=0.05.

|  |  | |  | |  | |  | |  |
| --- | --- | --- | --- | --- | --- | --- | --- | --- | --- |
| PCMCI connections | | |  | |  | |  | |  |
| Leader to follower | | |  | |  | |  | |  |
| Variable i | Variable j | | Time lag of i | | Link type i --- j | | Link value | |  |
| LFZ | FF4 | | 4 | | --> | | 0,015027 | |  |
| LF4 | FFZ | | 3 | | --> | | -0,015636 | |  |
| LF3 | FFZ | | 0 | | o-o | | 0,014845 | |  |
| LFZ | FFZ | | 0 | | o-o | | 0,015062 | |  |
| LFZ | FF4 | | 2 | | --> | | -0,020927 | |  |
| LFZ | FF4 | | 3 | | --> | | 0,019407 | |  |
| LF4 | FFZ | | 0 | | o-o | | 0,015381 | |  |
| LF4 | FF4 | | 0 | | o-o | | 0,017414 | |  |
| LF4 | FF4 | | 3 | | --> | | 0,01875 | |  |
| LFZ | FF3 | | 0 | | o-o | | 0,013694 | |  |
| LF3 | FF4 | | 5 | | --> | | 0,017571 | |  |
| LFZ | FF3 | | 3 | | --> | | 0,015002 | |  |
| LFZ | FF3 | | 4 | | --> | | -0,021779 | |  |
| LFZ | FF4 | | 2 | | --> | | 0,015088 | |  |
| LFZ | FF4 | | 4 | | --> | | -0,025561 | |  |
| LFZ | FF4 | | 5 | | --> | | 0,018318 | |  |
| LF3 | FF4 | | 2 | | --> | | 0,015517 | |  |
| LFZ | FF4 | | 2 | | --> | | -0,017624 | |  |
| LFZ | FF4 | | 3 | | --> | | -0,016169 | |  |
| LFZ | FF4 | | 4 | | --> | | 0,020224 | |  |
| LF4 | FF3 | | 5 | | --> | | 0,019644 | |  |
| Follower to leader | | | |  | |  | |  | |
| Variable i | | Variable j | | Time lag of i | | Link type i --- j | | Link value | |
| FF3 | | LFZ | | 3 | | --> | | 0,015767 | |
| FF3 | | LFZ | | 4 | | --> | | -0,016571 | |
| FFZ | | LF3 | | 5 | | --> | | 0,015128 | |
| FF3 | | LF3 | | 2 | | --> | | -0,018099 | |
| FF3 | | LF3 | | 5 | | --> | | -0,014814 | |
| FF3 | | LF4 | | 2 | | --> | | 0,015237 | |
| FF3 | | LF4 | | 5 | | --> | | -0,018493 | |
| FFZ | | LF3 | | 2 | | --> | | -0,016907 | |
| FF4 | | LF3 | | 2 | | --> | | 0,02341 | |
| FF4 | | LFZ | | 2 | | --> | | 0,018295 | |
| FF4 | | LF4 | | 5 | | --> | | 0,019573 | |
| FF3 | | LFZ | | 3 | | --> | | -0,020339 | |
| FFZ | | LF3 | | 3 | | --> | | -0,014658 | |

| Experiment # | Number of connections from L to F | Number of connections from F to L |  |
| --- | --- | --- | --- |
| 1 | 2 | 2 |  |
| 2 | 7 | 1 |  |
| 3 | 1 | 0 |  |
| 4 | 0 | 0 |  |
| 5 | 6 | 8 |  |
| 6 | 5 | 2 |  |
|  | 21 | 13 | In total |
|  | 3,5 | 2,16666667 | Mean |
|  | 2,62995564 | 2,73353658 | Std |
